# “I'm not too old to lift”: exploring lifelong involvement in Olympic weightlifting through the serious leisure perspective

**DOI:** 10.3389/fspor.2025.1483898

**Published:** 2025-06-16

**Authors:** François Gravelle, Aida Stratas, George Karlis

**Affiliations:** School of Human Kinetics, Faculty of Health Sciences, University of Ottawa, Ottawa, ON, Canada

**Keywords:** Olympic weightlifting, exercise commitment, aging, serious leisure, physical activity

## Abstract

**Introduction:**

This study explores the lifelong involvement of older adults in Olympic weightlifting (OW) with the aim of understanding the factors that motivate them to initiate and sustain participation across their lifespan, as well as the challenges they face and the benefits they experience.

**Methods:**

Semi-structured in-depth interviews were conducted with 22 participants (18 males, 4 females), aged 50–89, who had over 30 years of training experience in OW. The interviews lasted 40–58 min and were conducted face-to-face or via video call. The data was framed through Stebbins ’serious leisure perspective with an inductive thematic analysis to identify themes.

**Results and discussion:**

Five themes and four subthemes were identified that shape participants’ enduring involvement in OW: lifelong passion and commitment to OW (subthemes: perseverance and serious leisure career development), rigorous training regimens, injury experiences and recovery, self-improvement and personal growth, and social and community building (subthemes: community and camaraderie, coaching and mentorship for athlete development and legacy). These themes reveal the deep dedication, resilience, and strong sense of community that mark participants’ enduring involvement in this sport. The results suggest that OW can be utilized as a valuable sport for healthy aging, personal growth, and building supportive networks, which can inform approaches in health promotion, fitness programming, and sport development across different age groups.

## 1 Introduction. Olympic weightlifting: a sport for seniors

OW is a highly technical and physically rigorous sport that typically attracts younger adults. However, a dedicated minority of enthusiasts continue their involvement in this sport well into their later years of life despite life transitions, such as moving from education to a career, establishing a family, and balancing professional and personal responsibilities. The unique demands of Olympic weightlifting set it apart from other forms of resistance training. As suggested by the International Weightlifting Federation (1), OW is a sport involving two competitive events, the snatch and clean and the jerk event. The snatch involves lifting the barbell from the floor directly overhead in one movement. The clean and jerk involves lifting a barbell in two movements from the floor to the shoulders and then overhead. OW movements differ from other forms of exercises involving weights due to their complexity and need to have specific training and coaching to ensure the techniques are safely performed. These movements require specific coordination, timing, and joint mobility involving the use of various muscle groups. As athletes gain more experience, they can increase their total training volume (2). According to the literature, practicing OW in later life, whether at a competitive or recreational level, provides several advantages, including improved strength, bone density, flexibility, proprioception, explosiveness, and strong functional capacity for a better quality of life for older adults (3–6).

Aging or masters’ athletes, who often have decades of experience in OW, tend to endure unique exercise-related injuries and conditions due to their regular training regimens (7, 8). Common injuries include rotator cuff tears, Achilles tendinopathies, meniscal tears in the knee, as well as spine and shoulder injuries (8–10). Conditions such as osteoarthritis or chronic inflammation are also common among Olympic weightlifters (10). Coaches must be aware of these athletes’ particular issues and recognize when certain injuries and conditions might become detrimental. Tendinopathies and tendon ruptures are more prevalent in master athletes up to around 65 or 70 years-old, beyond which these injuries can worsen as muscle strength decreases faster than tendon strength (3, 11). Consequently, OW training programs should be tailored according to the age and physical abilities of the athletes with a gradual decrease in the amount of training as athletes move on with age, to prevent overtraining and reduce the risk of exercise-related injury (5, 12, 13). For older adults, motivators for OW include goal achievement, fitness, enjoyment, friendships, and preventing age-related declines (14, 15). However, misconceptions about becoming too muscular along with fear of injury and pain are common barriers among older adults taking on an OW training program (12). Thus, health professionals should emphasize the benefits of OW training as a powerful tool to improving muscle mass, strength, coordination, and the overall physical functioning of the body, and encourage participation among older adults (3, 5, 12).

Many psychological benefits are associated to the regular practice of OW. Nevertheless, there are a gaps in the literature understanding motivations, sustained engagement factors, and adaptive strategies used by lifelong Olympic weightlifters. Additionally, little is known about the specific challenges they face and how they overcome them. The purpose of this research is to address these gaps. Therefore, this study aims to fill these gaps by exploring the enduring involvement of older adults in OW and offers a detailed examination of their experiences, motivations, benefits, and challenges through the Serious Leisure Perspective (SPL) theoretical framework. To help narrow this gap in the existing core of research regarding the lifelong involvement of Olympic weightlifter in their sport, this research report will provide a brief review of literature, detailed information on the method followed to gather information, a results section, followed by discussion, limitations of the research, implications for future research leading to a general conclusion section.

## 2 Lifelong involvement in Olympic weightlifting (OW): a literature review

Older adults participate in competitive sports and physical activities for various reasons beyond maintaining physical health. Older adults are mostly motivated by the establishment of personal challenges, the competition experience, social connectivity, and opportunities for travel, all of which contribute to an enriched lifestyle in later stages of life (14). Masters’ sports can provide and maintain older individuals with athletic identities, foster a sense of empowerment and self-efficacy through the maintenance or redefinition of capabilities in later life (14). Older adults experience broader benefits from their involvement in competitive sports and physical activity that extend beyond physical activity, providing them namely with psychological and social advantages.

Among active older adults, there are distinct views on successful aging between men and women. Men are more inclined to see competitive athletes and active individuals as role models. Women tend to adopt a more holistic and inclusive view of aging (16). High physical function is often associated with successful aging. This belief may enhance societal pressures that may overlook alternative, equally beneficial ways of successful aging (17, 18). Whether Masters athletes serve as ideal models of successful aging or whether they contribute to unrealistic expectations that may not be applicable to all older adults is still at the heart of an ongoing debate (16).

Zhou, Yajun and Tian (19), argue that regular physical activity was identified as a pivotal factor in the optimization of the aging process, offering numerous benefits to elderly individuals. It has been observed to enhance physical capacities, such as strength, flexibility, and autonomy, while also prompting a shift in life perspectives. Studies have demonstrated a positive correlation between regular physical activity and significant physical and mental health benefits in the elderly (19, 20). Sustained participation in physical activity allows older people to improve their skills, strengthen their sense of identity within their training community, and engage in a continuous process of personal growth (19). These aspects contribute to enhancing the quality of life of the elderly and preserving their autonomy. Furthermore, serious leisure sports play a pivotal role in fostering intergenerational cohesion and collective commitment. Participation in these structured sporting communities fosters a strong sense of belonging and mutual support, facilitating meaningful social interactions that mitigate feelings of isolation and boost self-esteem (21–23).

The intersection between serious recreational sport and active ageing is manifested through four critical thematic dimensions: physiological enhancement, cultivation of positive emotional states, reinforcement of optimistic life orientations, and facilitation of meaningful social engagement. The availability of structured leisure opportunities for older adults has made recreational sport a central mechanism for promoting active ageing (19).

Consistent with Stebbins’ (24) perspective on serious leisure, older adults who engage in competitive and highly structured sporting activities exhibit six defining characteristics: persistence, personal effort, professional commitment, enduring benefits, formation of a strong identity, and adherence to a distinctive ethos (19, 25, 26). Through sustained participation, older adults improve their technical skills, cultivate strong group bonds and engage in a continuous process of personal development, accumulating significant physiological and psychosocial benefits (19).

In addition, serious recreational sport is an essential socio-cultural framework that fosters intergenerational cohesion and collective participation. The structured nature of these sporting communities facilitates the formation of deep social bonds, strengthens the sense of belonging, and enhances social cohesion (19, 20).

Research on the training habits of older weightlifters is limited but steadily increasing.

Studies indicate that older athletes often adapt their training regimens to accommodate the physical changes associated with biological declines and avoid injuries related to training.

A recent study by Huebner, Meltzer, Wenjuan, and Arrow (27) found that older weightlifters maintained high levels of muscle strength and power through regular and intensive training involving sessions of 1–2 h practiced from 2 to 4 days per week. This training regimen, however, was shown to increase their risk of injury compared to less active older adults. Despite this finding by Huebner et al. (27), the authors found that the injury rates among older weightlifters revealed to be lower than those observed in other sports. Injury associated with weightlifting also depends on the participant's skills and techniques, which affect their injury risk. For instance, Fragala et al. (28) observed that older weightlifters who prioritize correct training techniques and positioning over heavy or maximal weight lifts can avoid or reduce their risk of injury.

The SLP provides a useful framework for understanding the motivations behind lifelong involvement in OW. According to Stebbins (29), serious leisure is characterized by “the systematic pursuit of an amateur, hobbyist, or volunteer activity that participants find so substantial, interesting, and fulfilling that they launch themselves on a (leisure) career centered on acquiring and expressing its special skills, knowledge, and experience” (p. 5). Results by Stratas, Karlis, Gravelle, and Lagace's (30) study on older adults’ serious involvement in weightlifting, found that participants were motivated by intrinsic factors such as a passion for learning and mastering complex techniques as well as challenging their bodies and pushing their performance to new heights. The social aspect of weightlifting, such as camaraderie and a supportive community within weightlifting clubs, aligns with Stebbins’ notion of lasting benefits. These include social rewards and personal growth, which enhance long-term commitment.

Furthermore, research demonstrates that enduring involvement in OW provides numerous physical, psychological, and social benefits for older adults. Physically, weightlifting helps maintain muscle mass, strength, and bone density, and reduce the risk of developing sarcopenia and osteoporosis (31). Psychological benefits include enhanced mood, lower instances of depression, and improved self-confidence. Self-confidence particularly was found to be critically important as individuals tend to lose confidence with age due to age-related declines, health weaknesses, and ageism (30, 32). Socially, weightlifting creates a sense of belonging and connection to the sport community, which is crucial to avoiding social isolation and loneliness in older age (33). These benefits reflect the personal and social rewards outlined in the SLP, which demonstrate the crucial value of serious involvement in leisure activities like weightlifting.

Despite the benefits, lifelong weightlifters face several challenges. Physical challenges include slower recovery times, increased risk of injury, and the need to continuously modify training regimen to accommodate declining physical abilities (34). Psychologically, maintaining motivation can be difficult, especially when facing physical decline or plateauing performance. Moreover, societal stereotypes and ageism can discourage older adults from continuing in a sport that is perceived as the territory of the young (35). The SLP acknowledges that serious leisure participants often confront challenges that require perseverance, dedication, and adaptive strategies to maintain their involvement over time.

The SLP by Stebbins (24) offers a useful framework for understanding what motivates individuals to commit to long-term involvement in a leisure activity such as OW. According to the SLP framework, there are six qualities that determine this commitment: (1) perseverance to maintain participation despite failures and setbacks, (2) applying continuous personal effort to acquire special skills and experience, (3) leisure career that shows progression over time, (4) durable benefits such as personal enrichment and deep satisfaction, (5) strong identification with the activity pursued, and (6) unique ethos, which involves the social world or subculture associated with the activity. This study applies the SLP framework to explain why and how older adults maintain long-term involvement in weightlifting for decades, despite encountering adversity that may otherwise prevent them from continuing participation over their life-course. While the existing literature provides valuable insights into the general benefits of weightlifting for older adults, it largely overlooks the specific experiences of those who have engaged in OW over their lifetimes.

## 3 Research methods

Our study adopted an interpretive stance, focusing on the lived experiences of Olympic weightlifters and the meanings they attribute to their activities (36). By focusing on their personal experiences and subjective interpretations, we aim to gain a deeper understanding of how these participants perceive and make sense of their long- term commitment to weightlifting. This approach allows us to explore the motivations, challenges, and rewards that shape their involvement in the sport, providing rich, qualitative insights into their lived experiences.

### 3.1 Instrument

Semi-structured, in-depth qualitative interviews were conducted using an interview questionnaire structured into five key sections to comprehensively explore participants’ experiences in Olympic Weightlifting (OW). The first section gathered general background information, including daily living habits (e.g., “*Can you talk about your daily living habits? What are some of the challenges that you encounter in connection to your daily living habits related, namely to work, family and health…*?”*).* The second section examined participants’ engagement in physical activity throughout their life course (e.g., “*Can you talk about your experiences/history of physical activity in general?*”). The third section focused on their involvement in OW, incorporating questions based on Stebbins’ (24) six serious leisure characteristics, such as perseverance (e.g., “*Have you encountered adversity during your career, and how have you managed to overcome it?*”). The fourth section explored constraints, facilitators, and negotiation strategies, addressing barriers to participation and coping mechanisms (e.g., “*What factors hinder your involvement in OW, and what strategies do you employ to overcome these challenges?*”). The final section evaluated OW as a structured program. It investigated key motivational factors for attracting new participants (e.g., “What do you consider the most important factors in encouraging newcomers to participate in OW?”). This structured approach helped develop a nuanced understanding of the factors that shape athletes’ engagement in OW namely within the framework of serious leisure.

### 3.2 Participants

Semi-structured, in-depth qualitative interviews were conducted with a total of 22 Olympic weightlifters, including 18 males and 4 females, aged between 50 and 89 years (Mean age = 69.5 ± 19.5). The interviews, lasting between 40 and 58 min, were carried out either face-to-face or remotely through video calls.

Participants were recruited based on a purposive sampling method namely at Masters weightlifting competitions and at regular international weightlifting competitions, through referrals. As inclusion criteria participants needed to be 50 years of age and older, affiliated with a Weightlifting association, and having a minimum of 30 years of training experience in Olympic weightlifting. The average number of years of experience was 46.5, with a range of 52 years. The distribution of training experience spanned from a minimum of 27 years to a maximum of 77 years. Exclusion criteria included younger participants, newcomers to the sport of OW and individuals who were involved in resistance training other than OW. This study was conducted with the approval of the Research Ethics Board at the authors’ university to ensure adherence to ethical standards and guidelines. Informed consent was obtained from participants, and confidentiality ensured through detailed discussions and signed forms.

### 3.3 Study design

The researchers developed an interview guide for this qualitative study, drawing on Høffding and Martiny (37) and Siegenthaler and O'Dell's (38) approaches. The questions were designed based on Stebbins's (24, 29) six qualities of serious leisure: perseverance, personal effort, leisure career, identification, durable benefits, and unique ethos. For instance, under “perseverance,” participants described how they began participation in OW and sustained their physical training across their lifetime despite life transitions and setbacks. The goal was to assess participants’ commitment and motivation to adopting and maintaining long-term participation in OW.

### 3.4 Data analysis

Inductive thematic analysis, following Braun and Clarke's (39) six-phase process of reflective analysis, was used to identify and report themes. The six phases such as familiarization with data, coding, generating themes, reviewing themes, defining and naming themes, and writing the findings were applied rigorously. Interviews were audio-recorded, transcribed, and participants were anonymized with pseudonyms. The data analysis was conducted collaboratively, without the use of software in collaboration with one of the main researchers leading the process and receiving regular input and feedback from coauthors to ensure they are interpreting and categorizing the data in a consistent manner to enhance the reliability and validity of the identified themes and reduce subjective bias. During the coding phase, all transcripts were carefully reviewed, and a codebook was developed to maintain consistency in identifying key concepts. Codes were grouped into themes through multiple iterations, guided by repeated patterns and shared meanings across the data. The process involved not just identifying surface-level (semantic) elements, but also interpreting deeper (latent) meanings. Themes were iteratively developed and refined through several rounds of discussion, to ensure that they accurately captured the data. Redundant or overlapping themes were removed. To enhance the credibility and trustworthiness of the findings, the final themes were reviewed by an independent expert in qualitative research and were verified against the original dataset. These themes, along with supporting extracts from participant interviews, are presented in the results section.

## 4 Results

The analysis of our study, framed by Stebbins’ SLP (24, 29), revealed five overarching themes and four subthemes that led to involvement in OW across participants’ life-course. the first theme, lifelong passion and commitment, included subthemes such as the development of a serious leisure career and perseverance, which stressed the strong dedication and long-term involvement of participants. The second theme, rigorous training regimens across life, emphasized the discipline required to maintain OW practice over the years. The third theme, injury experiences and recovery strategies, focused on the challenges athletes faced with injuries and how they adapted and recovered. The fourth theme, self-improvement and personal growth, illustrated how participants used OW to strengthen not only their physical bodies but also other aspects of their lives. The final theme, social and community building, featured subthemes of community and camaraderie as well as coaching and mentorship for athlete development and legacy, revealing the importance of social connections and the role of mentorship in promoting both personal and community growth within the world of OW. Each theme and its corresponding subthemes are explored in detail and supported by excerpts from participants’ accounts. Table 1 shows the themes and subthemes, along with a sample quote that illustrates the insights shared by participants.

**Table 1:** The table shows the themes and subthemes and quote samples that illustrate the insights shared by participants.

Theme	Subtheme	Sample quotes
1. Lifelong passion and commitment to OW	a) Development of a serious leisure career	*“I started lifting when I was 17 in a little gym with friends, but it quickly became a passion of mine. I went from casual training to competing in regional, national, and international events. I don't compete as much anymore though, but I still train almost every day. It keeps me strong and active.” (James)*
	b) Perseverance	*“I did struggle to balance my family, work and OW training. It was very tough. But I love it! I can't quit just because of that. It actually gives me the strength to handle my problems better. So, I keep training no matter what. It's been training for over 40 years now.” (Martha)*
2. Rigorous training regimens across life		*“I'm a nurse but I structure my life where I train every single day. I have to convince myself sometimes… hey it's okay to take a day off. It's a struggle that I have.” (Melanie)*
3. Injury experiences and recovery strategies		*“Weightlifting has been good for me. I've had worst injuries in terms of playing hockey. I trained with a lot of other Olympic weightlifter, and for the most part they’ve been mostly healthy because we use the proper techniques and give enough time to recover between training.” (Ernie)*
4. Self-improvement and personal growth		*“My training is not just about lifting heavier weights. It's about constantly challenging my body and abilities and improve to become a better version of myself.” (Jake)*
5. Social and community building	a) Community and camaraderie	*“The community of Olympic weightlifters is incredible. You can rely on them for advice and learn from one another.” (Mike)*
	b) Coaching and mentorship for athlete development and legacy	*“I coached at club level, provincial level, at the national and as a national team head coach. I continue very actively to shadow, teach, and monitor coaches, athletes’ clubs and officials and participate in promoting Olympic Weightlifting.” (Gil)*

### 4.1 Theme 1: lifelong passion and commitment

Participants demonstrated a profound and enduring passion for OW since youth, which was critical in fostering their long-term commitment to the sport.

#### 4.1.1 Development of a serious leisure career

Most participants initiated their involvement in OW during their late teens or early adulthood, embarking on a serious leisure career characterized by consistent and rigorous training. They discussed starting out as beginners and gradually advancing their skills and techniques and competing at different stages and levels throughout their life. This commitment facilitated their progression throughout various stages and levels within the sport, ultimately leading to significant experience and mastery in OW as they aged. James exemplifies this stating:

I started lifting when I was 17 in a little gym with friends, but it quickly became a passion of mine. I went from casual training to competing in regional, national, and international events. I don’t compete as much anymore though, but I still train almost every day. It keeps me strong and active.

Similarly, Debra remarked, “Olympic Weightlifting goes all the way back when I found a good coach back in the 80s. I fell in love with it and kept at it since. I started as a beginner and progressed as the years went on. I still compete today.” Tom explained, “when I first got into OW, I had no idea it would become such a big part of my life. It started as a hobby in my early twenties and turned into something I took very seriously. I've been training and competing for decades now.”

Romeo has been involved in OW since 1948, including being on the national team until 1975 stating: “I've been lifting since I was a teenager. I've never stopped. OW has been a constant part of my life. Even after retiring from the national team in 75, I continued to train. It's about discipline and passion. At 76, I still train because this sport is part of who I am.”

#### 4.1.2 Perseverance

Olympic weightlifters sustained participation in the sport despite facing various adversities. At 71 years-old, Adam, for example, has maintained a rigorous training regimen despite his advancing age, stating: “I train 5 days a week, and it's some pretty mean training. Mondays and Tuesdays are snatch, Tuesdays and Wednesdays are clean and jerk, and I do between 250 and 300 weightlifting movements. But sometimes I feel pain when I train because I'm getting old, but I learned to work around it.”

Martha emphasized her perseverance stating, “There were times when I injured myself badly and wanted to quit, especially now that I'm older. But I love OW so much. It's always been part of me. I've been doing it for over 30 years. I just adapt and keep going.”

Alex, “I did struggle to balance my family, work and OW training. It was very tough. But I love it! I can’t quit just because of that. It actually gives me the strength to handle my problems better. So, I keep training no matter what. It's been training for over 40 years now.”

These participants exemplify how a deep-seated passion for OW drives lifelong commitment and fosters perseverance despite experiencing setbacks.

### 4.2 Theme 2: rigorous training regimens across life

A hallmark of the participants’ involvement in OW is their adherence to a consistent and rigorous training schedule, maintained over decades.

Participants discussed training four to six days a week, reflecting significant personal effort and unwavering commitment. This disciplined approach underscores the serious leisure nature of their involvement in OW. All spoke of the rigorous and consistent training schedules of 4–7 days a week that they currently maintain, and have sustained since youth, which shows the significant personal effort, commitment and longevity qualities of serious leisure participants.

Melanie articulated the challenges of maintaining such a training regimen: “I'm a nurse but I structure my life where I train every single day. I have to convince myself sometimes… hey it's okay to take a day off. It's a struggle that I have.” Sean indicated that “I've been training 6 days a week ever since I was 17, and even now in my sixties, it hasn’t changed. If I miss my training, I wouldn’t feel like myself.” While Faris shared, “I'm still following the same routine from way back in my twenties. I train almost every day and take a day off or 2 a week at most. Of course, I can’t lift as much weight as I used to, I'm 71 now, but I keep at it.”

### 4.3 Theme 3: injury experiences and recovery strategies

Despite the inherent risks associated with OW, participants managed injuries effectively, reinforcing their dedication to the sport.

Participants acknowledged common injuries resulting from their regular training but employed various strategies to manage and mitigate these risks. Their proactive approach to injury management demonstrates their commitment to maintaining long-term health and participation in OW. Participants highlighted the importance of using the correct techniques and recovery:

Ernie, “weightlifting has been good for me. I've had worst injuries in terms of playing hockey. I trained with a lot of other Olympic weightlifter, and for the most part they’ve been mostly healthy because we use the proper techniques and give enough time to recover between training.” Similarly, Bert stated “I have been doing weightlifting since the age of 18, for the past 67 years.

Now I lift very light weights. I still do it because I apply the right skills that I learned from years of practicing and give my body the enough rest to avoid getting injured.” While Alfred discussed modifying his training to minimize injury: “I focus more on flexibility and recovery now and I make sure that I listen to my body. I use strategies that I know to prevent injuring myself while doing my training.”

Tina discussed adapting her training to manage knee problems: “As I've gotten older, I've had to adjust my weightlifting training because of my knees. I had to use techniques that reduce pressure and stress on my knees and incorporate more recovery time. My strategy is if I do nothing, my knee problem will get worse. I can’t stop weightlifting. It's an important part of me! I just work around it.”

Participants perceived the long-term health benefits of OW as outweighing the risks of injury. Their commitment to safety practices, such as adhering to proper techniques and incorporating recovery periods, minimized injury risks and sustained their involvement.

### 4.4 Theme 4: self-improvement and personal growth

Participants reported deriving a deep sense of satisfaction when engaging in OW, which offers both a challenge, personal meaning, and fulfillment. They all discussed the broader benefits that arose from lifelong involvement in OW. These benefits stemmed from both their involvement in their respective clubs and in competitions.

Participants described OW as a powerful vehicle for personal development and growth. They reported experiencing a deep sense of development, achievement, and transformation that extended beyond simply their physical training and into all aspects of their lives. As the following participants explained:

“My training is not just about lifting heavier weights. It's about constantly challenging my body and abilities and improve to become a better version of myself.” (Jake)

“Weightlifting was an environment where I had an endless desire to better myself over the years, and become a great coach, and work with great people that I train because I saw weightlifting as means to an end, to attain a sense of accomplishment and strengthen my body and mind.” (Mike)

“When I first started in OW, I could barely lift the bar and didn’t know what I was doing, but over time I built my skills and a strong body that I'm proud of. This also made me stronger mentally because the discipline and perseverance that I built carries over into everything I do.” (Noah)

“Weightlifting is very meaningful. It makes difficult things in life more manageable. It makes me more resilient. If I didn’t have that to go to, it would be difficult to deal with the chaos and problems in my life.” (Luc)

### 4.5 Theme 5: social and community building

The social dimension of OW played a critical role in fostering a sense of community and camaraderie among participants, which increased their attachment to the sport and enhanced their OW experience. This sense of community and camaraderie that often started at club level often extended to provincial, national and often international levels based on the level of participation of the respondents. This sense of camaraderie and community could also be tainted by the different roles participants played in OW. Roles such as athletes, coaches or officials would open the possibility of being part of a variety of communities.

#### 4.5.1 Community and camaraderie

Participants valued the shared experiences and support networks within the OW community, which provided a sense of belonging and mutual support. This is illustrated in participant's accounts:

“Being part of this OW association gave me a support system that I could rely on, not just for working out but for making friendships. Sometimes we go out together for a beer after a heavy training session and talk about it and have fun and laugh.” (Kai)

“I feel like I'm part of something bigger. Of course there are always people that you don’t get along with, but for the most part it's a great community.” (Drew)

“The community of Olympic weightlifters is incredible. You can rely on them for advice and learn from one another. Knowing that I have similar struggles and wins in weightlifting as others is comforting to me. We help each other, and that makes me feel part of a special community.” (Mike)

#### 4.5.2 Coaching and mentorship for athlete development and legacy

Many participants have taken on coaching or mentorship roles to contribute to the continuity and growth of Olympic weightlifting, fostering a sense of legacy and community within the sport.

This is evident in participants’ narratives:

“I coached at club level, provincial level, at the national and as a national team head coach. I continue very actively to shadow, teach, and monitor coaches, athletes’ clubs and officials and participate in promoting Olympic Weightlifting.” (Gil)

“I coached a lot of athletes over the years. I also travel often to evaluate new OW trainers. I'm a judge for national and international OW competitions. In fact, I helped write some of the OW rules and guidelines.” (Marc)

“That's how I got into OW. I had an incredible coach when I was a teen who showed me how to train without destroying my body. After over 30 years of doing this, I'm now doing the same, I show young people how to use the right strategies to be better weightlifters. I love OW and want that legacy to continue that's why I coach.” (Greg)

“Coaching young athletes allows me to give back and pass what I learned on to others to keep this sport going.” (Debra)

**Figure 1:**
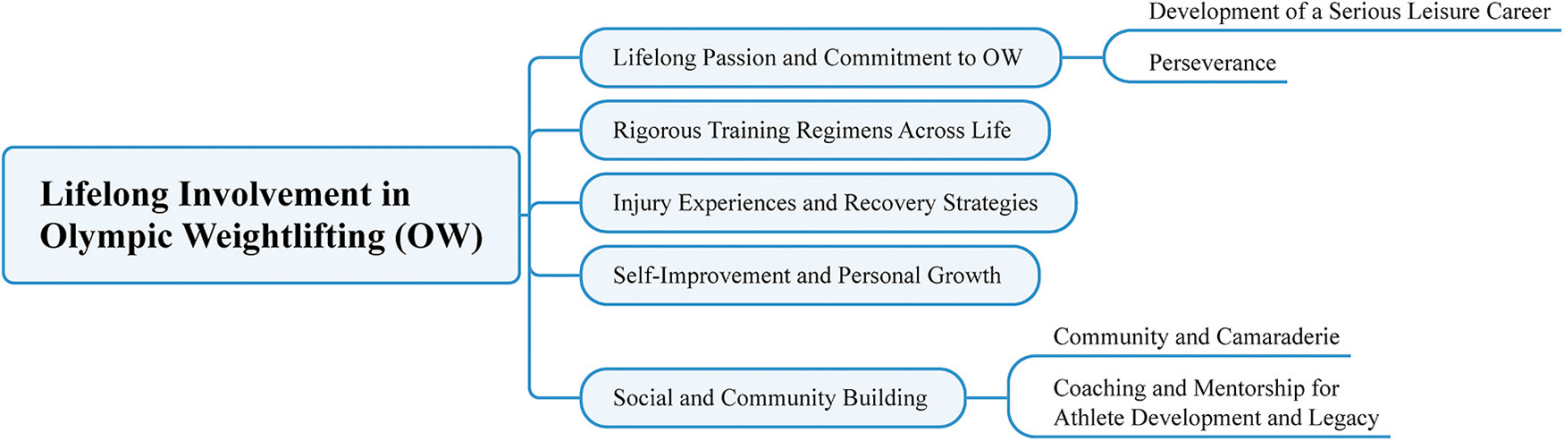
Mapping of research themes and subthemes.

Figure 1 illustrates the thematic map outlining the main themes and subthemes identified through data analysis.

## 5 Discussion

The results of this study highlight the deep commitment older adults have towards OW, framed within Stebbins’ SLP (24, 29). The themes and subthemes identified reveal the complex yet rewarding nature of enduring involvement in OW. Participants’ lifelong passion towards OW was found to have a direct correlation with commitment to sustain participation across the lifespan (40). In addition, our findings determined that OW provided participants with a lifelong tool that not only enabled them to set and achieve goals, improve their technical skills/performance, and build physical strength and endurance, but also strengthened their mental resilience to deal with challenges associated with everyday life more effectively. Our age study has also shown OW to be an effective means to resist aging, providing participants with positive perspectives of their aging bodies. Most appreciate what their fit bodies can still do, even at an advanced age. This finding coincides with Dionigi's (41) research, which explored the motives and experiences of master athletes over the age of 60 involved in physically demanding sports. Dionigi found that their serious sport participation challenged conventional norms in and fostered a sense of pride in being fit and more active than their lesser and non-active peers.

This sense of fulfillment and commitment to sport participation resonates with Stebbins’ notion of serious leisure, which frames lifelong involvement in an activity, whether in sport or otherwise, is seen as a personal career marked by continuous learning and personal growth, and a determination to overcome adversity, regardless of age. Despite life transitions and physical and mental challenges experienced by participants, participants displayed intense perseverance in maintaining participation across their lifespan, which reflects the resilience and commitment of serious leisure participants in OW. This correlates with Stratas et al. (30) and Webb, Stratas, and Karlis’ (32) findings who identified three categories of older exercisers: low, moderate, and high. Those in the high exercise category perceived their ongoing participation in a sport as a central life interest and managed to 'stick’ to their sport activity despite facing setbacks and failures along the way, including age-related challenges. The consistency in training regimens, with rigorous schedules of 4–6 days a week, underscores substantial personal effort and dedication. This regular and intensive training is essential for maintaining high physical strength and performance, particularly as one ages, as noted by Huebner et al. (27).

Complementing this point of view, Ronkainen, Kavoura, and Ryba (42) emphasize the role of athletic identity in nourishing motivation, sports commitment, and self-esteem. However, this identity can also involve psychological vulnerabilities, particularly during transitional phases, such as injury recovery or retirement. These authors also caution that when athletic identity becomes overly exclusive—serving as the individual's primary or sole source of self-esteem—athletes may become particularly susceptible to psychological distress. Thus, literature suggests that while serious leisure and strong athletic identity foster resilience and sustained engagement, they may also expose individuals to risks when identity becomes too rigid and focusing on limited sport-related elements.

Furthermore, participants ability to overcome constraints such as life transitions and age-related declines highlights the high level of adaptability and determination these older participants possess and contributes important insights to our understanding of what drives individuals to maintain enduring participation in a sport well into old age. Moreover, managing injury risks from training is critical for older Olympic weightlifters. While participants acknowledged the risks of developing injuries from their ongoing participation in OW, they stressed the significant health benefits and disease prevention that the sport offered. Put differently, the risk of injury from long-term participation was considered less significant than the risk of developing age-related diseases from lack of activity or sport participation.

They highlighted that adherence to safety practices during training is critical to avoiding or minimizing injuries and optimizing the health benefits of OW throughout their lifespan. Indeed, participants reported that Olympic weightlifters tend to sustain fewer injuries than participants in contact sports. Lower injury rates compared to other sports suggest proper training techniques and taking reasonable precautions to control risks are crucial for sustaining lifelong engagement in sport, as found by Karlsson and Rosengren (31), McKean, Manson, and Stanish (43), and Patelia, El-Bakri, Adli and Baker (44).

This underscores the importance of proper education, skilled coaching, and a solid support system to ensure the correct use of OW techniques, which are essential to decreasing injury risks. It also highlights the resilience and deep commitment of Olympic weightlifters in overcoming challenges associated with their lifelong participation in a demanding sport like OW, as it shows the ability of these participants to push through adversity such as injuries, weaknesses due to age-related declines, and intense training to maintain participation over the years. Moreover, the social and community aspects of weightlifting are pivotal for lifelong involvement in OW. The sense of camaraderie, support networks, and shared values within the weightlifting community align with the SLP's concept of a unique ethos and social world. Contributions to coaching and mentorship roles foster a sense of legacy and enhance the OW community's continuity and growth. This highlights the social rewards and personal enrichment derived from the sport, which ¨are crucial for avoiding social isolation and loneliness in older age, as noted by Grant (33).

## 6 Limitations

While this study provides valuable insights into the phenomenon of lifelong participation of Olympic weightlifters, there are several limitations that must be acknowledged. Firstly, the small sample size of 22 participants (18 males and 4 females), while it is adequate and common in qualitative research, may limit the representativeness to the broader population of Olympic weightlifters and other groups involved in allied sport-related activities such as weightlifting and bodybuilding. Therefore, as it is the case for qualitative research protocols, the results could not be generalized to late bloomers or late adherents this relatively small and predominantly male sample may not fully capture the diversity of experiences across different demographic groups. Accordingly, the generalizability of the findings to the wider population is limited.

However, the aim of this research study was not to generalize the data but rather to explore the deep, lived experiences of a rare and unique group of individuals who have extensive expertise in OW because of their decades of involvement in the sport. As such, the researchers focused on in-depth qualitative insights to allow for a rich, context-specific understanding of long-term commitment to OW. Secondly, due to the highly specialized nature of the study participants, being lifelong Olympic weightlifters with decades of experience, the findings may not fully apply to those who are new to the OW sport. While participants’ extensive experience provided unique and rich data, future research studies might benefit from exploring the experiences of broader or more diverse participant groups to complement this study's current findings.

Finally, the gender imbalance in the sample (with a predominance of male participants) could affect the interpretation of the results. While efforts were made to recruit female participants, the underrepresentation of women may have resulted in insights that reflect more the experiences and perspectives of men than woman. Future research could explore gender-specific experiences to gain a more balanced view of lifelong participation in Olympic weightlifting. Despite these limitations, the study provides rich, qualitative insights that contribute meaningfully to understanding the motivations, challenges and benefits of lifelong Olympic weightlifters, which can serve as a foundation for future research in the field.

## 7 Implications for future research

This study, being qualitative and interpretive in nature, raises potential opportunities for future research. Firstly, while the study contributes to a deeper understanding of why older adults initiate and maintain participation in OW across their lifespan, as well as the benefits and challenges derived from their lifelong participation, future studies could incorporate quantitative methods to further validate and expand on the findings. For example, quantitative research could employ larger and more diverse samples to examine and compare the similarities and differences between various demographic groups, such as age, gender, and experience levels, and in terms of their motivations for participating in OW, as well as the benefits and challenges they experience. This would provide a greater understanding of how these factors vary across different populations.

Secondly, opportunities in conducting longitudinal studies could provide precious information on how motivations and experiences in OW evolve over time, and how participants adapt their training to accommodate declines in physical capacities as they age. Tracking these changes over time would provide critical insights into how older Olympic weightlifters modify their regimens, techniques, and goals to maintain participation in the sport across their life-course despite the challenges faced by their aging bodies. Such research could uncover important strategies on how lifelong participants balance maintaining performance across time with preventing injuries, thus offering valuable insights into long-term athletic sustainability.

Thirdly, mixed-methods research could be used to incorporate both qualitative and quantitative data. This would provide a more holistic view of the experiences of lifelong OW participants. By combining both qualitative insights and quantitative data, future studies could provide a richer understanding of the complex factors underlying the motivations, challenges and benefits of long-term participation in OW.

Fourthly, it would be interesting in future research to further study those who decided to stop their involvement in OW.

Despite these limitations, this study offers several important strengths. The qualitative nature of this study as framed by the SLP sheds light on a little-explored topic and a unique population group where existing research is limited. This study increases understanding of the factors driving commitment and motivations to long-term involvement in OW, and reveals the challenges and the personal and social benefits derived from sustained participation in OW across time (24, 29, 38). Moreover, the data derived from this study can inform the design of tailored interventions and support systems aimed at promoting lifelong participation in the OW sport. The findings have practical implications for health professionals, coaches, and policymakers in promoting lifelong involvement in OW. By understanding the unique motivations and challenges/benefits of older Olympic weightlifters, tailored training programs, and support systems can be developed to promote their longevity and wellbeing in the sport.

## 8 Conclusion

The enduring involvement of older adults in OW, as viewed through the SLP, emphasizes the sport's challenging yet fulfilling, and community-oriented nature. The physical and mental demands of the sport was found to push participants to continuously refine and perfect their skills, while the structured training regimens provided a sense of progression. Despite the challenging nature of the sport, weightlifters found fulfillment in their personal growth, strong physical and mental health, and the satisfaction of mastering complex techniques.

In addition, Olympic weightlifting was found to foster a strong sense of community and strengthen social bonds among participants. This environment encouraged participants to rely on one another, share their experiences, and build camaraderie, which reinforced their attachment to sport. Furthermore, the study's findings highlight the importance of having passion for a sport, which was shown to drive participants to continue practicing Olympic weightlifting over the years. This passion fueled their commitment, which helped them maintain consistent training despite setbacks such as life transitions and injuries related to OW. Ultimately, their passion for OW played a crucial role in sustaining their lifelong involvement and dedication to the sport. These findings suggest that fostering passion, community bonding, and skill development in sport or physical activity can promote lifelong participation, particularly among older adults. Future research could explore how these insights might be applied to other sports or physical activities to encourage long-participation and improve well-being in older populations. To this end, practitioners, such as coaches and health professionals, can harness this knowledge to develop tailored programs that enhance motivation, minimize sport-injury risks, and build supportive communities to sustain lifelong involvement in sport or physical activities.

## Data Availability

The raw data supporting the conclusions of this article will be made available by the authors, without undue reservation.
